# Crystal structure of {[1′-(di­phenyl­phosphino)ferrocen­yl]meth­yl}di­methyl­ammonium chloride monohydrate

**DOI:** 10.1107/S2056989017013408

**Published:** 2017-09-25

**Authors:** Martin Zábranský, Petr Štěpnička

**Affiliations:** a Department of Inorganic Chemistry, Faculty of Science, Charles University, Hlavova 2030, 128 40 Prague 2, Czech Republic

**Keywords:** crystal structure, ferrocene, amines, phosphines, structure elucidation

## Abstract

Individual ions and the solvating water mol­ecule constituting the structure of the title compound, [Fe(C_8_H_13_N)(C_17_H_14_P)]Cl·H_2_O, assemble into dimeric units located around crystallographic inversion centers *via* N—H⋯Cl and O—H⋯Cl hydrogen bonds. These discrete fragments are further inter­connected into chains by C—H⋯O inter­actions. The disubstituted ferrocene core in the {[1′-(di­phenyl­phosphino)ferrocen­yl]meth­yl}di­methyl­ammonium cation has an approximate synclinal eclipsed conformation and is tilted by 3.40 (11)°.

## Abstract 2

The mol­ecular structure of {[1′-(di­phenyl­phosphino)ferrocen­yl]meth­yl}di­methyl­ammonium chloride monohydrate is presented. Individual ions and the solvating water mol­ecule assemble into dimeric units located around crystallographic inversion centers *via* N—H⋯Cl and O—H⋯Cl hydrogen bonds.

## Chemical context   

Chiral phosphinoferrocene amines are recognized to be efficient supporting ligands for transition-metal-catalysed reactions as well as useful synthetic precursors for a range of ferrocene derivatives (Štěpnička *et al.*, 2008 ▸). In contrast, their non-chiral counterparts have received limited attention. While studying functional derivatives of the ubiquitous 1,1′-bis­(di­phenyl­phosphino)ferrocene (dppf), we have devised an alternative synthesis of 1′-(di­phenyl­phosphino)-1-[(di­methyl­amino)­meth­yl]ferrocene, Ph_2_PfcCH_2_NMe_2_ (fc = ferrocene-1,1′-di­yl), firstly reported by Wright (1990 ▸), and studied this compound as a ligand in Pd^II^ and Au^I^ complexes (Štěpnička *et al.*, 2012 ▸). More recently, we have converted this phosphino­amine into a phosphinoferrocene betaine Ph_2_PfcCH_2_NMe_2_(CH_2_)_3_SO_3_, which was in turn used to prepare new functional ferrocene phosphines (Zábranský *et al.*, 2015 ▸, 2017 ▸). This contribution describes the crystal structure of a hydrated hydro­chloride of this amine, [Ph_2_PfcCH_2_NHMe_2_]Cl·H_2_O, which was isolated serendipitously while regenerating the amine after preparation of the aforementioned betaine.
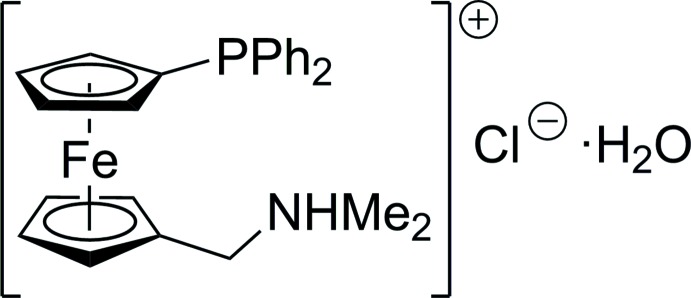


## Structural commentary   

**Figure 1:**
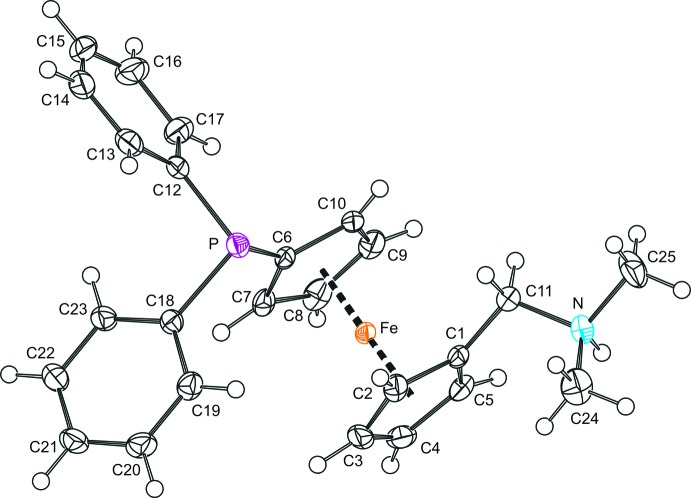
*PLATON* (Spek, 2009 ▸) plot of the cation in the structure of the title compound. Displacement ellipsoids correspond to the 50% probability level.

A view of the mol­ecular structure of the title compound, with atom labelling, is shown in Fig. 1 ▸. The ferrocene moiety in the {[1′-(di­phenyl­phosphino)ferrocen­yl]meth­yl}di­methyl­ammonium cation has a regular geometry with the individual Fe—C bonds ranging from 2.0239 (15) Å (C1) to 2.0489 (15) Å (C7). Its cyclo­penta­dienyl rings are tilted by 3.40 (11)° and assume an eclipsed conformation with the attached substituents oriented in a synclinal fashion, as demonstrated by the torsion angle C1—*Cg*1—*Cg*2—C6 of −85.38 (12)°, where *Cg*1 and *Cg*2 are the centroids of the cyclo­penta­dienyl rings C1–C5 and C6–C10, respectively.

The protonated amino­methyl chain is directed away from the ferrocene core, with the angle between the C1—N bond and the axis of the ferrocene unit, *Cg*1⋯*Cg*2, being 148.99 (11)°. The phosphine substituent at the other cyclo­penta­dienyl ring is oriented so that one of its pivotal P—C(Ph) bonds lies nearly in the plane of the bonding five-membered ring C6–C10, while the other is roughly parallel with the axis of the ferrocene unit. The angle at which the P—C18 bond inter­sects the C6–C10 plane is 13.17 (10)°, whereas the angle subtended by the P—C12 bond and the *Cg*1⋯*Cg*2 line is only 8.68 (5)°.

## Supra­molecular features   

**Figure 2:**
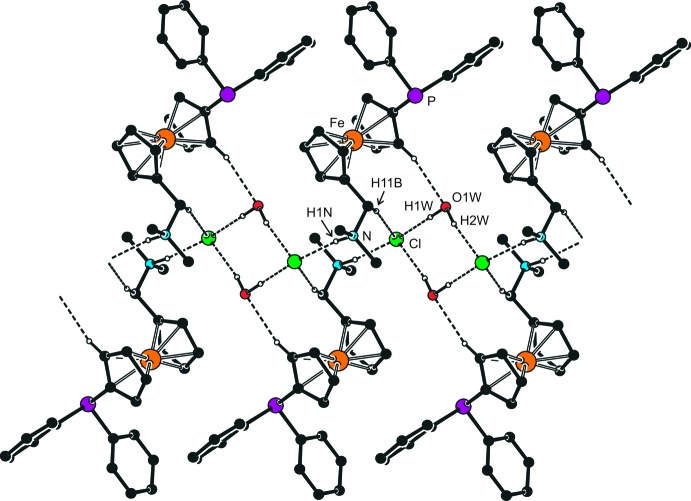
Section of the hydrogen-bonded chains in the structure of the title compound. For clarity, hydrogen atoms not involved in hydrogen bonding are omitted.

Each [Ph_2_PfcCH_2_NHMe_2_]^+^ cation in the structure of the title compound is involved in an N—H⋯Cl hydrogen bond to a proximal chloride anion (for hydrogen-bond parameters, see Table 1 ▸). The anions further act as hydrogen-bond acceptors for a pair of inversion-related water mol­ecules, which in turn results in the formation of charge-neutral, closed dimeric arrays {(Ph_2_PfcCH_2_NHMe_2_)_2_Cl_2_(H_2_O)_2_} around the crystallographic inversion centers. These discrete units are further inter­linked into chains along the *a* axis *via* the weaker C—H⋯O and C—H⋯Cl inter­actions, as shown in Fig. 2 ▸.

## Database survey   

A search in the Cambridge Structural Database (Version 5.38 with the latest update from May 2017; Groom *et al.*, 2016 ▸) for structurally related compounds resulted in the structures of two similar (ferrocenylmeth­yl)ammonium salts, namely *N*-(ferrocenylmeth­yl)di­methyl­ammonium chloride (Winter & Wolmershäuser, 1998 ▸) and its dihydrate (Guo *et al.*, 2006 ▸), and two complexes obtained from Ph_2_PfcCH_2_NMe_2_ featuring a protonated (di­methyl­amino)­methyl side chain, *viz*. [AuCl(Ph_2_PfcCH_2_NHMe_2_)]*X*, where *X* = Cl and ClO_4_ (Štěpnička *et al.*, 2012 ▸).

## Synthesis and crystallization   

The ‘amine’ Ph_2_PfcCH_2_NMe_2_ regenerated from the synthesis of the phosphinoferrocene betaine Ph_2_PfcCH_2_NMe_2_(CH_2_)_3_SO_3_ (Zábranský *et al.*, 2015 ▸) (*ca* 100 mg) was dissolved in acetic acid (10 mL) and the solution was evaporated under reduced pressure. After this procedure was repeated twice using chloro­form as a solvent, the residue was dissolved in a minimum amount of hot ethyl acetate. The solution was filtered and layered with hexane. Crystallization by liquid-phase diffusion over several days afforded orange crystals of the title compound. The yield was not determined.

Analysis calculated for [C_25_H_27_FeNP]Cl·H_2_O (481.76 g mol^−1^): C 62.32, H 6.07, N 2.91%. Found: C 62.23, H 5.91, N 2.79%. ESI MS: *m*/*z* 383 ([Ph_2_PfcCH_2_]^+^), 428 ([Ph_2_PfcCH_2_NMe_2_ + H]^+^)

## Refinement   

Relevant crystallographic data and structure refinement parameters are summarized in Table 2 ▸. All non-hydrogen atoms were refined freely with anisotropic displacement parameters. The hydrogen atoms of the water mol­ecule and the NH proton were located on a difference electron-density map and refined as riding atoms with *U*_iso_(H) set to 1.2*U*_eq_ of their bonding atom. Hydrogen atoms bonded to carbons were included in their theoretical positions and refined as riding atoms with *U*_iso_(H) = 1.2*U*_eq_(C).

## Supplementary Material

Crystal structure: contains datablock(s) global, I. DOI: 10.1107/S2056989017013408/im2484sup1.cif

Structure factors: contains datablock(s) I. DOI: 10.1107/S2056989017013408/im2484Isup3.hkl

CCDC reference: 1575391

Additional supporting information:  crystallographic information; 3D view; checkCIF report

## Figures and Tables

**Table 1:** Hydrogen-bond geometry (Å, °)

*D*—H⋯*A*	*D*—H	H⋯*A*	*D*⋯*A*	*D*—H⋯*A*
N—H1*N*⋯Cl	0.92	2.13	3.0323 (16)	167
O1*W*—H1*W*⋯Cl	0.98	2.23	3.2162 (19)	177
O1*W*—H2*W*⋯Cl^i^	1.02	2.29	3.289 (2)	166
C10—H10⋯O1*W* ^ii^	0.95	2.46	3.390 (3)	165
C11—H11*B*⋯Cl^ii^	0.99	2.77	3.7369 (17)	167

Symmetry codes: (i) 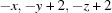; (ii) 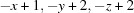.

**Table 2:** Experimental details

Crystal data	
Chemical formula	[Fe(C_8_H_13_N)(C_17_H_14_P)]Cl·H_2_O
*M*_r_	481.76
Crystal system, space group	Triclinic, *P* 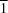
Temperature (K)	150
*a*, *b*, *c* (Å)	7.9888 (3), 12.7596 (6), 13.2311 (5)
α, β, γ (°)	111.037 (1), 104.075 (1), 99.628 (2)
*V* (Å^3^)	1171.76 (8)
*Z*	2
Radiation type	Mo *K*α
μ (mm^−1^)	0.84
Crystal size (mm)	0.27 × 0.26 × 0.14

Data collection	
Diffractometer	Bruker D8 VENTURE Kappa Duo PHOTON 100 CMOS
Absorption correction	Numerical (*SADABS*; Bruker, 2014 ▸)
*T*_min_, *T*_max_	0.78, 0.89
No. of measured, independent and observed [*I* > 2σ(*I*)] reflections	24764, 5361, 4820
*R*_int_	0.024
(sin θ/λ)_max_ (Å^−1^)	0.650

Refinement	
*R*[*F* ^2^ > 2σ(*F* ^2^)], *wR*(*F* ^2^), *S*	0.028, 0.074, 1.06
No. of reflections	5361
No. of parameters	273
H-atom treatment	H-atom parameters constrained
Δρ_max_, Δρ_min_ (e Å^−3^)	0.77, −0.53

Computer programs: *Instrument Service* and *SAINT* (Bruker, 2015 ▸), *SHELXT* (Sheldrick, 2015*a* ▸), *SHELXL2014* (Sheldrick, 2015*b* ▸) and *PLATON* (Spek, 2009 ▸).

## supplementary crystallographic information

### Crystal data

**Table:**

[Fe(C_8_H_13_N)(C_17_H_14_P]Cl·H_2_O	*Z* = 2
*M**_r_* = 481.76	*F*(000) = 504
Triclinic, *P*1	*D*_x_ = 1.365 Mg m^−^^3^
*a* = 7.9888 (3) Å	Mo *K*α radiation, λ = 0.71073 Å
*b* = 12.7596 (6) Å	Cell parameters from 9875 reflections
*c* = 13.2311 (5) Å	θ = 2.7–27.5°
α = 111.037 (1)°	µ = 0.84 mm^−^^1^
β = 104.075 (1)°	*T* = 150 K
γ = 99.628 (2)°	Plate, orange
*V* = 1171.76 (8) Å^3^	0.27 × 0.26 × 0.14 mm

### Data collection

**Table:**

Bruker D8 VENTURE Kappa Duo PHOTON 100 CMOS diffractometer	5361 independent reflections
Radiation source: IµS micro-focus sealed tube	4820 reflections with *I* > 2σ(*I*)
Quazar Mo multilayer optic monochromator	*R*_int_ = 0.024
φ and ω scans	θ_max_ = 27.5°, θ_min_ = 2.7°
Absorption correction: numerical (SADABS; Bruker, 2014)	*h* = −10→10
*T*_min_ = 0.78, *T*_max_ = 0.89	*k* = −16→16
24764 measured reflections	*l* = −17→15

### Refinement

**Table:**

Refinement on *F*^2^	Primary atom site location: structure-invariant direct methods
Least-squares matrix: full	Secondary atom site location: difference Fourier map
*R*[*F*^2^ > 2σ(*F*^2^)] = 0.028	Hydrogen site location: mixed
*wR*(*F*^2^) = 0.074	H-atom parameters constrained
*S* = 1.06	*w* = 1/[σ^2^(*F*_o_^2^) + (0.0302*P*)^2^ + 0.8395*P*] where *P* = (*F*_o_^2^ + 2*F*_c_^2^)/3
5361 reflections	(Δ/σ)_max_ < 0.001
273 parameters	Δρ_max_ = 0.77 e Å^−^^3^
0 restraints	Δρ_min_ = −0.53 e Å^−^^3^

### Special details

**Table:**

Geometry. All esds (except the esd in the dihedral angle between two l.s. planes) are estimated using the full covariance matrix. The cell esds are taken into account individually in the estimation of esds in distances, angles and torsion angles; correlations between esds in cell parameters are only used when they are defined by crystal symmetry. An approximate (isotropic) treatment of cell esds is used for estimating esds involving l.s. planes.

### Fractional atomic coordinates and isotropic or equivalent isotropic displacement parameters (Å^2^)

**Table:**

	*x*	*y*	*z*	*U*_iso_*/*U*_eq_	
Fe	0.35629 (3)	0.59451 (2)	0.78521 (2)	0.01686 (7)	
P	0.60918 (5)	0.42728 (3)	0.65913 (3)	0.02008 (9)	
N	0.48959 (19)	0.94040 (11)	0.77917 (12)	0.0222 (3)	
H1N	0.4115	0.9619	0.8185	0.027*	
C1	0.3623 (2)	0.73548 (13)	0.74808 (13)	0.0190 (3)	
C2	0.3012 (2)	0.63030 (14)	0.64396 (13)	0.0246 (3)	
H2	0.3559	0.6120	0.5860	0.029*	
C3	0.1436 (2)	0.55845 (15)	0.64331 (15)	0.0305 (4)	
H3	0.0755	0.4832	0.5850	0.037*	
C4	0.1056 (2)	0.61835 (16)	0.74453 (17)	0.0306 (4)	
H4	0.0075	0.5903	0.7655	0.037*	
C5	0.2396 (2)	0.72753 (15)	0.80907 (15)	0.0242 (3)	
H5	0.2463	0.7853	0.8805	0.029*	
C6	0.5294 (2)	0.49253 (13)	0.77727 (13)	0.0188 (3)	
C7	0.3773 (2)	0.44264 (14)	0.80049 (13)	0.0214 (3)	
H7	0.2959	0.3668	0.7549	0.026*	
C8	0.3684 (3)	0.52571 (16)	0.90340 (14)	0.0282 (4)	
H8	0.2794	0.5153	0.9380	0.034*	
C9	0.5155 (3)	0.62697 (15)	0.94565 (14)	0.0295 (4)	
H9	0.5427	0.6956	1.0139	0.035*	
C10	0.6153 (2)	0.60790 (14)	0.86822 (14)	0.0240 (3)	
H10	0.7199	0.6617	0.8753	0.029*	
C11	0.5289 (2)	0.83286 (14)	0.79066 (14)	0.0231 (3)	
H11A	0.6098	0.8067	0.7468	0.028*	
H11B	0.5921	0.8529	0.8723	0.028*	
C12	0.7610 (2)	0.35513 (13)	0.71926 (14)	0.0218 (3)	
C13	0.8462 (2)	0.28984 (15)	0.64905 (17)	0.0315 (4)	
H13	0.8258	0.2852	0.5736	0.038*	
C14	0.9599 (2)	0.23210 (16)	0.6890 (2)	0.0422 (5)	
H14	1.0132	0.1856	0.6396	0.051*	
C15	0.9964 (3)	0.24150 (17)	0.7998 (2)	0.0442 (6)	
H15	1.0754	0.2022	0.8268	0.053*	
C16	0.9178 (3)	0.30812 (18)	0.8711 (2)	0.0395 (5)	
H16	0.9444	0.3160	0.9479	0.047*	
C17	0.7991 (2)	0.36410 (16)	0.83049 (16)	0.0290 (4)	
H17	0.7439	0.4088	0.8797	0.035*	
C18	0.4138 (2)	0.30073 (13)	0.56095 (13)	0.0194 (3)	
C19	0.2952 (2)	0.31205 (15)	0.47127 (14)	0.0257 (3)	
H19	0.3154	0.3842	0.4641	0.031*	
C20	0.1473 (2)	0.21872 (16)	0.39223 (15)	0.0303 (4)	
H20	0.0669	0.2278	0.3321	0.036*	
C21	0.1172 (2)	0.11288 (16)	0.40097 (15)	0.0293 (4)	
H21	0.0170	0.0491	0.3465	0.035*	
C22	0.2337 (2)	0.10042 (15)	0.48951 (15)	0.0298 (4)	
H22	0.2135	0.0278	0.4957	0.036*	
C23	0.3807 (2)	0.19393 (14)	0.56956 (14)	0.0249 (3)	
H23	0.4590	0.1848	0.6306	0.030*	
C24	0.3962 (3)	0.91775 (17)	0.65899 (16)	0.0363 (4)	
H24A	0.3688	0.9890	0.6563	0.054*	
H24B	0.2841	0.8547	0.6282	0.054*	
H24C	0.4738	0.8945	0.6128	0.054*	
C25	0.6568 (3)	1.03899 (16)	0.83233 (19)	0.0405 (5)	
H25A	0.7412	1.0191	0.7907	0.061*	
H25B	0.7117	1.0534	0.9126	0.061*	
H25C	0.6277	1.1095	0.8288	0.061*	
Cl	0.23009 (6)	1.03869 (4)	0.89357 (4)	0.02978 (10)	
O1W	0.0368 (2)	1.16218 (15)	1.07177 (16)	0.0569 (4)	
H2W	−0.0268	1.1016	1.0938	0.068*	
H1W	0.0973	1.1233	1.0189	0.068*	

### Atomic displacement parameters (Å^2^)

**Table:**

	*U*^11^	*U*^22^	*U*^33^	*U*^12^	*U*^13^	*U*^23^
Fe	0.01965 (11)	0.01487 (11)	0.01374 (11)	0.00601 (8)	0.00136 (8)	0.00560 (8)
P	0.0215 (2)	0.01765 (19)	0.01958 (19)	0.00385 (15)	0.00531 (15)	0.00777 (15)
N	0.0266 (7)	0.0174 (6)	0.0224 (7)	0.0044 (5)	0.0097 (6)	0.0076 (5)
C1	0.0216 (7)	0.0165 (7)	0.0189 (7)	0.0076 (6)	0.0027 (6)	0.0089 (6)
C2	0.0342 (9)	0.0206 (8)	0.0164 (7)	0.0094 (7)	0.0009 (6)	0.0092 (6)
C3	0.0296 (9)	0.0221 (8)	0.0272 (8)	0.0013 (7)	−0.0102 (7)	0.0117 (7)
C4	0.0178 (8)	0.0319 (9)	0.0445 (10)	0.0069 (7)	0.0042 (7)	0.0225 (8)
C5	0.0243 (8)	0.0242 (8)	0.0298 (8)	0.0141 (7)	0.0101 (7)	0.0137 (7)
C6	0.0212 (7)	0.0152 (7)	0.0183 (7)	0.0073 (6)	0.0021 (6)	0.0071 (6)
C7	0.0282 (8)	0.0188 (7)	0.0214 (7)	0.0090 (6)	0.0078 (6)	0.0122 (6)
C8	0.0408 (10)	0.0328 (9)	0.0202 (8)	0.0186 (8)	0.0127 (7)	0.0160 (7)
C9	0.0409 (10)	0.0271 (9)	0.0147 (7)	0.0170 (8)	−0.0003 (7)	0.0052 (6)
C10	0.0226 (8)	0.0178 (7)	0.0222 (8)	0.0067 (6)	−0.0048 (6)	0.0056 (6)
C11	0.0216 (8)	0.0201 (7)	0.0261 (8)	0.0070 (6)	0.0051 (6)	0.0091 (6)
C12	0.0160 (7)	0.0162 (7)	0.0273 (8)	0.0016 (6)	0.0043 (6)	0.0059 (6)
C13	0.0202 (8)	0.0249 (8)	0.0353 (10)	0.0023 (7)	0.0078 (7)	−0.0001 (7)
C14	0.0194 (8)	0.0228 (9)	0.0659 (15)	0.0055 (7)	0.0101 (9)	0.0010 (9)
C15	0.0200 (9)	0.0268 (9)	0.0811 (17)	0.0074 (7)	0.0050 (9)	0.0245 (10)
C16	0.0291 (10)	0.0403 (11)	0.0527 (12)	0.0103 (8)	0.0038 (9)	0.0296 (10)
C17	0.0266 (9)	0.0312 (9)	0.0334 (9)	0.0122 (7)	0.0091 (7)	0.0170 (8)
C18	0.0216 (7)	0.0187 (7)	0.0158 (7)	0.0062 (6)	0.0057 (6)	0.0051 (6)
C19	0.0323 (9)	0.0243 (8)	0.0205 (8)	0.0114 (7)	0.0060 (7)	0.0094 (6)
C20	0.0301 (9)	0.0345 (9)	0.0199 (8)	0.0128 (7)	−0.0004 (7)	0.0083 (7)
C21	0.0227 (8)	0.0279 (9)	0.0238 (8)	0.0040 (7)	0.0010 (7)	0.0018 (7)
C22	0.0286 (9)	0.0223 (8)	0.0298 (9)	0.0014 (7)	0.0014 (7)	0.0092 (7)
C23	0.0247 (8)	0.0229 (8)	0.0222 (8)	0.0040 (6)	0.0001 (6)	0.0101 (7)
C24	0.0516 (12)	0.0342 (10)	0.0265 (9)	0.0102 (9)	0.0105 (8)	0.0188 (8)
C25	0.0382 (11)	0.0231 (9)	0.0487 (12)	−0.0046 (8)	0.0140 (9)	0.0083 (8)
Cl	0.0340 (2)	0.0292 (2)	0.0265 (2)	0.01592 (17)	0.01079 (17)	0.00835 (17)
O1W	0.0473 (9)	0.0474 (9)	0.0704 (12)	0.0122 (8)	0.0307 (9)	0.0117 (8)

### Geometric parameters (Å, º)

**Table:**

Fe—C1	2.0239 (15)	C9—H9	0.9500
Fe—C5	2.0341 (16)	C10—H10	0.9500
Fe—C10	2.0389 (16)	C11—H11A	0.9900
Fe—C8	2.0411 (16)	C11—H11B	0.9900
Fe—C9	2.0433 (16)	C12—C17	1.386 (2)
Fe—C2	2.0433 (16)	C12—C13	1.401 (2)
Fe—C4	2.0469 (17)	C13—C14	1.384 (3)
Fe—C3	2.0472 (16)	C13—H13	0.9500
Fe—C6	2.0472 (15)	C14—C15	1.380 (3)
Fe—C7	2.0489 (15)	C14—H14	0.9500
P—C6	1.8084 (16)	C15—C16	1.377 (3)
P—C12	1.8390 (17)	C15—H15	0.9500
P—C18	1.8427 (16)	C16—C17	1.397 (2)
N—C24	1.480 (2)	C16—H16	0.9500
N—C25	1.485 (2)	C17—H17	0.9500
N—C11	1.508 (2)	C18—C23	1.395 (2)
N—H1N	0.9207	C18—C19	1.396 (2)
C1—C5	1.426 (2)	C19—C20	1.393 (2)
C1—C2	1.437 (2)	C19—H19	0.9500
C1—C11	1.487 (2)	C20—C21	1.383 (3)
C2—C3	1.424 (3)	C20—H20	0.9500
C2—H2	0.9500	C21—C22	1.385 (2)
C3—C4	1.418 (3)	C21—H21	0.9500
C3—H3	0.9500	C22—C23	1.394 (2)
C4—C5	1.421 (2)	C22—H22	0.9500
C4—H4	0.9500	C23—H23	0.9500
C5—H5	0.9500	C24—H24A	0.9800
C6—C7	1.428 (2)	C24—H24B	0.9800
C6—C10	1.440 (2)	C24—H24C	0.9800
C7—C8	1.421 (2)	C25—H25A	0.9800
C7—H7	0.9500	C25—H25B	0.9800
C8—C9	1.420 (3)	C25—H25C	0.9800
C8—H8	0.9500	O1W—H2W	1.0206
C9—C10	1.424 (3)	O1W—H1W	0.9824
			
C1—Fe—C5	41.14 (6)	C7—C6—P	128.31 (12)
C1—Fe—C10	106.92 (7)	C10—C6—P	124.39 (13)
C5—Fe—C10	126.23 (7)	C7—C6—Fe	69.66 (9)
C1—Fe—C8	148.86 (7)	C10—C6—Fe	69.05 (8)
C5—Fe—C8	115.03 (7)	P—C6—Fe	127.25 (8)
C10—Fe—C8	68.79 (7)	C8—C7—C6	108.38 (15)
C1—Fe—C9	115.70 (7)	C8—C7—Fe	69.38 (9)
C5—Fe—C9	104.86 (7)	C6—C7—Fe	69.53 (8)
C10—Fe—C9	40.82 (7)	C8—C7—H7	125.8
C8—Fe—C9	40.70 (8)	C6—C7—H7	125.8
C1—Fe—C2	41.36 (6)	Fe—C7—H7	126.9
C5—Fe—C2	69.00 (7)	C9—C8—C7	108.19 (15)
C10—Fe—C2	119.30 (7)	C9—C8—Fe	69.73 (9)
C8—Fe—C2	167.52 (7)	C7—C8—Fe	69.97 (9)
C9—Fe—C2	151.52 (8)	C9—C8—H8	125.9
C1—Fe—C4	69.01 (7)	C7—C8—H8	125.9
C5—Fe—C4	40.74 (7)	Fe—C8—H8	126.0
C10—Fe—C4	163.98 (7)	C8—C9—C10	108.27 (15)
C8—Fe—C4	106.22 (8)	C8—C9—Fe	69.57 (9)
C9—Fe—C4	125.71 (8)	C10—C9—Fe	69.43 (9)
C2—Fe—C4	68.59 (7)	C8—C9—H9	125.9
C1—Fe—C3	69.10 (6)	C10—C9—H9	125.9
C5—Fe—C3	68.56 (7)	Fe—C9—H9	126.7
C10—Fe—C3	154.05 (8)	C9—C10—C6	107.85 (15)
C8—Fe—C3	128.15 (8)	C9—C10—Fe	69.75 (9)
C9—Fe—C3	164.68 (8)	C6—C10—Fe	69.67 (9)
C2—Fe—C3	40.75 (7)	C9—C10—H10	126.1
C4—Fe—C3	40.52 (8)	C6—C10—H10	126.1
C1—Fe—C6	129.28 (6)	Fe—C10—H10	126.1
C5—Fe—C6	166.26 (7)	C1—C11—N	112.09 (13)
C10—Fe—C6	41.28 (6)	C1—C11—H11A	109.2
C8—Fe—C6	68.83 (7)	N—C11—H11A	109.2
C9—Fe—C6	68.93 (6)	C1—C11—H11B	109.2
C2—Fe—C6	110.24 (7)	N—C11—H11B	109.2
C4—Fe—C6	152.74 (7)	H11A—C11—H11B	107.9
C3—Fe—C6	120.44 (7)	C17—C12—C13	118.37 (16)
C1—Fe—C7	168.72 (6)	C17—C12—P	123.83 (13)
C5—Fe—C7	149.81 (7)	C13—C12—P	117.78 (14)
C10—Fe—C7	68.83 (7)	C14—C13—C12	120.41 (19)
C8—Fe—C7	40.66 (6)	C14—C13—H13	119.8
C9—Fe—C7	68.44 (7)	C12—C13—H13	119.8
C2—Fe—C7	130.66 (6)	C15—C14—C13	120.62 (19)
C4—Fe—C7	117.98 (7)	C15—C14—H14	119.7
C3—Fe—C7	109.84 (7)	C13—C14—H14	119.7
C6—Fe—C7	40.81 (6)	C16—C15—C14	119.70 (18)
C6—P—C12	100.79 (7)	C16—C15—H15	120.1
C6—P—C18	101.23 (7)	C14—C15—H15	120.1
C12—P—C18	100.82 (7)	C15—C16—C17	120.0 (2)
C24—N—C25	111.96 (15)	C15—C16—H16	120.0
C24—N—C11	112.50 (13)	C17—C16—H16	120.0
C25—N—C11	110.50 (14)	C12—C17—C16	120.80 (18)
C24—N—H1N	105.9	C12—C17—H17	119.6
C25—N—H1N	107.8	C16—C17—H17	119.6
C11—N—H1N	107.9	C23—C18—C19	118.43 (15)
C5—C1—C2	107.59 (15)	C23—C18—P	123.76 (12)
C5—C1—C11	124.99 (15)	C19—C18—P	117.78 (12)
C2—C1—C11	127.30 (15)	C20—C19—C18	120.71 (16)
C5—C1—Fe	69.82 (9)	C20—C19—H19	119.6
C2—C1—Fe	70.04 (9)	C18—C19—H19	119.6
C11—C1—Fe	122.55 (11)	C21—C20—C19	120.27 (16)
C3—C2—C1	107.63 (15)	C21—C20—H20	119.9
C3—C2—Fe	69.77 (9)	C19—C20—H20	119.9
C1—C2—Fe	68.59 (9)	C20—C21—C22	119.64 (16)
C3—C2—H2	126.2	C20—C21—H21	120.2
C1—C2—H2	126.2	C22—C21—H21	120.2
Fe—C2—H2	127.0	C21—C22—C23	120.31 (16)
C4—C3—C2	108.37 (15)	C21—C22—H22	119.8
C4—C3—Fe	69.73 (10)	C23—C22—H22	119.8
C2—C3—Fe	69.48 (9)	C22—C23—C18	120.63 (15)
C4—C3—H3	125.8	C22—C23—H23	119.7
C2—C3—H3	125.8	C18—C23—H23	119.7
Fe—C3—H3	126.6	N—C24—H24A	109.5
C3—C4—C5	108.16 (16)	N—C24—H24B	109.5
C3—C4—Fe	69.75 (10)	H24A—C24—H24B	109.5
C5—C4—Fe	69.14 (9)	N—C24—H24C	109.5
C3—C4—H4	125.9	H24A—C24—H24C	109.5
C5—C4—H4	125.9	H24B—C24—H24C	109.5
Fe—C4—H4	126.8	N—C25—H25A	109.5
C4—C5—C1	108.24 (15)	N—C25—H25B	109.5
C4—C5—Fe	70.11 (10)	H25A—C25—H25B	109.5
C1—C5—Fe	69.05 (9)	N—C25—H25C	109.5
C4—C5—H5	125.9	H25A—C25—H25C	109.5
C1—C5—H5	125.9	H25B—C25—H25C	109.5
Fe—C5—H5	126.5	H2W—O1W—H1W	107.9
C7—C6—C10	107.30 (14)		
			
C5—C1—C2—C3	−0.93 (17)	Fe—C9—C10—C6	−59.48 (10)
C11—C1—C2—C3	175.34 (14)	C8—C9—C10—Fe	58.89 (11)
Fe—C1—C2—C3	59.05 (11)	C7—C6—C10—C9	0.14 (17)
C5—C1—C2—Fe	−59.99 (11)	P—C6—C10—C9	−178.91 (11)
C11—C1—C2—Fe	116.29 (15)	Fe—C6—C10—C9	59.53 (11)
C1—C2—C3—C4	0.75 (18)	C7—C6—C10—Fe	−59.39 (10)
Fe—C2—C3—C4	59.06 (12)	P—C6—C10—Fe	121.56 (11)
C1—C2—C3—Fe	−58.32 (10)	C5—C1—C11—N	−77.55 (19)
C2—C3—C4—C5	−0.27 (19)	C2—C1—C11—N	106.78 (17)
Fe—C3—C4—C5	58.64 (11)	Fe—C1—C11—N	−164.53 (10)
C2—C3—C4—Fe	−58.91 (11)	C24—N—C11—C1	−59.33 (18)
C3—C4—C5—C1	−0.31 (18)	C25—N—C11—C1	174.72 (14)
Fe—C4—C5—C1	58.70 (11)	C6—P—C12—C17	−3.86 (16)
C3—C4—C5—Fe	−59.01 (12)	C18—P—C12—C17	−107.64 (15)
C2—C1—C5—C4	0.77 (17)	C6—P—C12—C13	177.80 (13)
C11—C1—C5—C4	−175.61 (14)	C18—P—C12—C13	74.02 (14)
Fe—C1—C5—C4	−59.36 (11)	C17—C12—C13—C14	2.5 (2)
C2—C1—C5—Fe	60.13 (10)	P—C12—C13—C14	−179.02 (14)
C11—C1—C5—Fe	−116.26 (15)	C12—C13—C14—C15	−2.5 (3)
C12—P—C6—C7	−89.13 (14)	C13—C14—C15—C16	0.6 (3)
C18—P—C6—C7	14.33 (15)	C14—C15—C16—C17	1.2 (3)
C12—P—C6—C10	89.71 (13)	C13—C12—C17—C16	−0.8 (3)
C18—P—C6—C10	−166.83 (13)	P—C12—C17—C16	−179.14 (14)
C12—P—C6—Fe	178.35 (9)	C15—C16—C17—C12	−1.0 (3)
C18—P—C6—Fe	−78.20 (11)	C6—P—C18—C23	−83.71 (15)
C10—C6—C7—C8	0.36 (17)	C12—P—C18—C23	19.72 (15)
P—C6—C7—C8	179.36 (12)	C6—P—C18—C19	97.97 (13)
Fe—C6—C7—C8	−58.65 (11)	C12—P—C18—C19	−158.60 (13)
C10—C6—C7—Fe	59.01 (10)	C23—C18—C19—C20	−0.1 (2)
P—C6—C7—Fe	−121.99 (12)	P—C18—C19—C20	178.34 (13)
C6—C7—C8—C9	−0.73 (18)	C18—C19—C20—C21	−0.7 (3)
Fe—C7—C8—C9	−59.47 (11)	C19—C20—C21—C22	0.7 (3)
C6—C7—C8—Fe	58.75 (11)	C20—C21—C22—C23	0.1 (3)
C7—C8—C9—C10	0.81 (18)	C21—C22—C23—C18	−0.8 (3)
Fe—C8—C9—C10	−58.81 (11)	C19—C18—C23—C22	0.8 (3)
C7—C8—C9—Fe	59.62 (11)	P—C18—C23—C22	−177.51 (14)
C8—C9—C10—C6	−0.58 (18)		

### Hydrogen-bond geometry (Å, º)

**Table:**

*D*—H···*A*	*D*—H	H···*A*	*D*···*A*	*D*—H···*A*
N—H1*N*···Cl	0.92	2.13	3.0323 (16)	167
O1*W*—H1*W*···Cl	0.98	2.23	3.2162 (19)	177
O1*W*—H2*W*···Cl^i^	1.02	2.29	3.289 (2)	166
C10—H10···O1*W*^ii^	0.95	2.46	3.390 (3)	165
C11—H11*B*···Cl^ii^	0.99	2.77	3.7369 (17)	167

Symmetry codes: (i) −*x*, −*y*+2, −*z*+2; (ii) −*x*+1, −*y*+2, −*z*+2.
